# Divergent Response to the SSRI Citalopram in Male and Female Three-Spine Sticklebacks (*Gasterosteus aculeatus*)

**DOI:** 10.1007/s00244-020-00776-1

**Published:** 2020-11-05

**Authors:** Martin Kellner, K. Håkan Olsén

**Affiliations:** School of Natural Sciences, Technology and Environmental Studies, Södertörn University, Alfred Nobels allé 7, 141 89 Huddinge, Sweden

## Abstract

Selective serotonin reuptake inhibitors (SSRIs) are psychotropic pharmaceuticals used as antidepressants. SSRIs are commonly found in surface waters in populated areas across the globe. They exert their effect by blocking the serotonin re-uptake transporter in the presynaptic nerve ending. The present study examined whether behavioural effects to exposure to SSRI citalopram depend on personality and sex in the stickleback (*Gasterosteus aculeatus*). Three aspects of stickleback behaviour are examined: feeding behaviour, aggression, and boldness. We exposed sticklebacks to 350–380 ng/l citalopram for 3 weeks. Feeding and aggressive behaviour were recorded before and after exposure, whereas scototaxis behaviour was tested after exposure. The results show treatment effects in feeding and aggressive behaviour. Feeding is suppressed only in the male group (*χ*^2^ = 20.4, *P* < 0.001) but not in the females (*χ*^2^ = 0.91, *P* = 0.339). Aggressive behaviour was significantly affected by treatment (*χ*^2^ = 161.9, *P* < 0.001), sex (*χ*^2^ = 86.3, *P* < 0.001), and baseline value (*χ*^2^ = 58.8, *P* < 0.001). Aggressiveness was suppressed by citalopram treatment. In addition, the fish showed no change in aggression and feeding behaviour over time regardless of sex and treatment, which indicate personality traits. Only females are affected by treatment in the scototaxis test. The exposed females spent significantly (*χ*^2^ = 5.02, *P* = 0.050) less time in the white zone than the female controls.

Ever since “Fish on Prozac” (Brooks et al. 2003), selective serotonin re-uptake inhibitors (SSRIs) have been an environmental concern across the globe. SSRIs are a class of antidepressants that exert their effect primarily by inhibiting the reuptake of serotonin into the presynaptic nerve ending, thus elevating the serotonin concentration in the synaptic cleft. Citalopram, which is used in this study, is generally considered the most selective SSRI on the market (Owens et al. 2001). The serotonergic system, which is the target of SSRIs, is phylogenetically ancient and largely conserved throughout the vertebrate phylum (Lillesaar 2011; Nowicki et al. 2014). Feeding, aggression, and boldness are behavioural traits that are all to some degree influenced by the serotonergic system. Administration of serotonin has a suppressing effect on feeding in organisms as diverse as goldfish (*Carassius auratus*) (De Pedro et al. 1998), rainbow trout (*Oncorhynchus mykiss*) (Ortega et al. 2013), rodents (Voigt and Fink 2015; Rozenblit-Susan et al. 2016), and ants (Falibene et al. 2012). Acute serotonin administration suppresses aggression in the fighting fish (*Betta splendens*) (Clotfelter et al. 2007). Strangely, reports of effects from the administration of serotonin on boldness in fish seem to be missing from literature, whereas reports of the behavioural effects of SSRIs are common (Kellner et al. 2016; Chiffre et al. 2016; Dzieweczynski et al. 2016; Egan et al. 2009; Giacomini et al. 2016). In stickleback, individuals that are aggressive against conspecifics also are bold (in contrast to shy), i.e., they show predatory inspection and risk-taking behaviour (Huntingford 1976; Bell and Stamps 2004; Bell and Sih 2007). The phenotypic correlation between aggressiveness and boldness is under genetic influence (Bakker 1994). The strength of the positive correlation between aggressiveness and boldness in three-spine stickleback is dependent on the predation pressure (Bell and Sih 2007). A strong positive correlation between aggression and boldness also was observed in zebrafish when five wild populations were studied, but there were population differences in the level of aggression and boldness (Martins and Bhat 2014). There was less variation in aggression and boldness within populations than across populations. No sites were found where fish were bold, but not aggressive, or fish were aggressive, but not bold; i.e., aggression and boldness are linked.

In accordance with their effects on serotonin reuptake, citalopram and other SSRIs have been shown to affect behaviours influenced by the serotonergic system. For example, exposure to both 0.15 μg/l and 1.5 μg/l of citalopram suppresses the feeding rate in the three-spine stickleback (Kellner et al. 2015) and exposure to 54 μg/l of fluoxetine reduces food intake and body weight in goldfish (Mennigen et al. 2009). Aggression in the fighting fish is reduced by 705 μg/l and 350 μg/l fluoxetine (Kohlert et al. 2012). Citalopram suppresses aggressive behaviour when administered orally to rainbow trout at a dose corresponding to 100 μg of citalopram/kg, but interestingly, just like the serotonin precursor tryptophan, only in individuals with experience of being dominant (Lepage et al. 2005). This may be due to subordinates already having high brain serotonin levels and high activity of the serotonergic system (Winberg et al. 1991). Behaviour related to stress response also is attenuated by citalopram in the three-spine stickleback (Kellner et al. 2016), zebrafish (*Danio rerio*) (Egan et al. 2009), and Endler guppy (*Poecilia wingei*) (Olsén et al. 2014). Citalopram-exposed three-spine stickleback interacted with a novel object significantly more often and for a longer time than control fish (Kellner et al. 2016). There were, however, no differences in the number of bites against its mirror image between the treatments.

Lately, personality (or temperament, Gosling 2001) in animals has become a focus of scientific literature (Weinstein et al. 2008; Carter et al. 2013; Sih et al. 2015). Animal personality often is defined as a behavioural pattern that is consistent over time and contexts (Gosling 2001; Carere and Eens 2005; Dingemanse and Réale 2005; Bell 2007; Herborn et al. 2010; Niemelä and Dingemanse 2014; Stamps and Biro 2016). Behavioural traits that are commonly measured are feeding (Silva et al. 2014), aggression (Thörnqvist et al. 2015), novel object inspection (Kellner et al. 2016), and behavioural as well as physiological stress response (Thörnqvist et al. 2015). Sometimes animal individuals are labelled as either reactive or proactive (Schjolden et al. 2005), where proactive individuals are typically bolder and less intimidated by stressful conditions, such as a novel environment. Correlations between multiple behavioural traits are known as behavioural syndromes (Huntingford 1976; Bell and Stamps 2004; Bell 2005; Carter et al. 2013). Recent studies indicate that proactive and reactive fish can respond differently to SSRI treatment (Fior et al. 2018).

In the current paper, we use the three-spine stickleback that have been used in several personality studies (Bell and Stamps 2004; Dingemanse et al. 2007) and many different behaviour studies to test the hypothesis that the SSRI pharmaceutical citalopram affects the behaviour of bold and shy fish differently. Furthermore, we hypothesise that males and females may be affected differently after citalopram exposures. We also tested whether behavioural syndromes exist in the stickleback.

## Materials and Methods

### Handling and Keeping of Fish

The three-spine stickleback (*Gasterosteus aculeatus*) is abundant in the northern hemisphere in freshwater, marine habitats, and brackish water, such as the Baltic Sea, where it has become very abundant (Eklöf et al. 2020). Three-spine sticklebacks were caught in Vikhögs harbour 29-km north of Malmö City (55°36′21″ N 13°02′09″ E) on the Swedish west coast on February 25, 2017 and transported to the stickleback facility at Stockholm University within 48 h. The fish were caught using a landing net with small mesh size. The fish were not infected by *Schistocephalus solidus,* which affects the brain monoaminergic system (Øverli et al. 2001) that affects the brain and behaviour. The fish were kept on a 8:16 day:night light regime. On September 12, the fish were transferred to the stickleback facility at Södertörn University where they were kept in two 300-l holding tanks. The water was aerated and circulated through a filter (EHEIM 2080 (1200 XL)) containing 2000 ml of active carbon (REEF-SPEC™ Carbon, Red Sea). The water temperature throughout the experiment was checked daily and stayed between 15 and 20 °C throughout the experiment. pH was checked once in two different samples during the course of the experiment and was 6.9. The fish were fed frozen bloodworms to satiation daily at 1 p.m. for 5 days per week. During weekends, they were fed freeze-dried bloodworms (JBL Novo Fil) by a feeder (Hydor). Faeces were removed from the holding tanks daily 5 days per week. The water lost while removing faeces was replenished from a water conditioning tank where the water was filtrated as in the other two tanks. The holding tank was refilled with tap-water every time any water was drawn from it and served as a primary filtration and to condition the water to room temperature.

### Experimental Procedures

The experiment was conducted with two batches of fish; each consisted of 30 fish. For each batch, the following procedure was used (Table 1). One fish was transferred from the holding tanks to each experimental aquarium (60 × 30 × 30 cm) and allowed to acclimatise for 7 days. On Days 8 and 9, baseline feeding and baseline aggressive behaviour, respectively, were recorded. On Day 12, a solution of citalopram dissolved in MilliQ water was added to every second aquarium to yield a nominal concentration of 1.5 μg/l. The aquaria acting as controls received a corresponding amount of MilliQ water. After 15 days of exposure, on Day 27, feeding behaviour was recorded. The day after, aggressive behaviour was recorded. On Day 34, the fish were put through a scototaxis test. The fish were sexed after the final scototaxis test. All behaviours were recorded on video camera and examined manually at a later point using the video software VLC media player (version 2.2.2 Weatherwax) running under Ubuntu Linux 16.04. The experimental aquaria contained a half clay pot for shelter and were cleaned every second day. During cleaning, half of the water volume was changed and replaced with water from the conditioning tank. Half the amount of the original dose of MilliQ water or citalopram solution was added to compensate for the amount lost. The experimental aquaria were lit from above with light racks (Sylvania Aquastar F30W/174-T8 10,000 K).

**Table 1:** Time table of tests performed with each fish

Day	Event
1	Fish are transferred to experimental aquaria
8	Baseline feeding is recorded
9	Baseline aggressive behaviour is recorded
12	Exposure starts
27	Feeding behaviour is recorded
27	Aggressive behaviour is recorded
34	Scototaxis behaviour is recorded
35	Anaesthesia in MS 222, measured, decapitated, and sexed

### Feeding Behaviour

Feeding behaviour was investigated by dropping a piece of frozen bloodworm into the fish home aquarium and recording feeding behaviour on video camera. When analysing, note was taken of latency to the first feeding strike and the number of feeding strikes during10 min. This method was previously employed in several studies (Kellner et al. 2015; 2018).

### Aggression

Aggression was tested by lowering a mirror into the fish home aquarium. Based on previous experience, the fish was given 5 min to acclimatise itself to the mirror. The number of attacks against the mirror image was then counted for the next 5 min. If the fish did not come out of hiding during the first 5 min, the fish was discarded from analysis. Stickleback aggressive behaviour is quite fierce, and it was considered a risk that the fish could hurt itself on the mirror. The procedure was chosen as a compromise between this risk and the need for sufficient time to get adequate data. The 5-minute timeframe was previously used for three-spine stickleback by others (Norton and Carreño Gutiérrez 2019).

### Scototaxis

The scototaxis test measures anxiety levels in fish (Caio et al. 2010). Three scototaxis aquaria were run in parallel, each recorded by a separate video camera. The scototaxis aquaria were fitted with white plastic covering the sides and bottom in half the aquarium and black plastic in the other half. In the middle was a transparent, easily removable compartment. Scototaxis was examined by gently transferring the fish from the home aquarium to the transparent compartment of a scototaxis aquarium. Each fish was given 5 min to acclimatise after which the transparent compartment was lifted, allowing the fish to choose side. Scototaxis was recorded for 10 min. The behaviour was analysed manually at a later point, and three variables were noted: the total time spent in the white half of the aquarium; the number of crossings to the white half, and the latency to the first cross to the white side.

### Water Sampling and Chemical Analyses

Water samples were taken at an interval of 2–3 days and from three aquaria each time. No aquarium was sampled more than once. The samples were analysed as follows. Ultrapure water was produced by a Milli-Q Advantage Ultrapure Water purification system and filtered through a 0.22-μm Millipak Express membrane and an LC-Pak^®^ polishing unit (Merk Millipore, Billercia, MA). Other chemicals, such as methanol, acetonitrile, and ammonium acetate, were all of high-performance liquid chromatography (HPLC)-grade and purchased from Sigma Aldrich (Sigma Aldrich, St. Louis, MO). The analytical standards used were of high purity (> 98%). The detailed information about analytical standards (Citalopram and Oxazepam-D5) has been provided elsewhere (Gago-Ferrero et al. 2017). For the two-dimensional liquid chromatography method coupled to tandem mass (LC/LC–MS/MS), duplicate (around 10-mL aliquots) thawed water samples were filtered through a syringe filter (0.22 μm, regenerated cellulose). All water samples were spiked with an internal standard of oxazepam-D5 to achieve a concentration of 50 ng/L. For LC/LC–MS/MS analysis, an LC/LC system from Thermo Fisher Scientific, San Jose, CA, was used for liquid chromatography. An Acquity UPLC BEH-C18 column (Waters, 100 mm × 2.1 i.d., 1.7 µm particle size from Waters Corporation, Manchester, UK) was used as an analytical column. A Hypersil GOLD aQ column (20 mm × 2.1 mm i.d., 12-µm particles, from Thermo Fisher Scientific, San Jose, CA) was used as an extraction column for online solid phase extraction. Injection volumes were 1.0 mL for all samples. A triple-stage quadrupole MS/MS TSQ Quantiva (Thermo Fisher Scientific) was used for the detection of Citalopram. Data were evaluated by using TraceFinder™ 3.3 software (Thermo Fisher).

### Statistics

Data were analysed as generalised linear models using the lme4 package v. 1.1–15 (Bates et al. 2015) in R v. 3.4.3 (R core team 2015). All response variables were analysed using a type II Wald *χ*^2^ test. In the analysis of scototaxis data, the testInteractions functions in the R phia package (version 0.2–1) was used for post hoc testing. For aggression and feeding data, treatment, sex, and baseline value were used as fixed factors in the initial analysis. For the scototaxis data, there was no baseline value, and thus sex and treatment were used. For count data, such as the number of feeding strikes or attacks against a mirror image, a Poisson distribution was applied. For time data, such as latency, a gaussian distribution was applied. Data were transformed when needed to meet normal distribution requirements. Correlations between aggression and feeding behaviour were examined by using Pearson’s product moment correlation coefficient.

## Results

### Feeding

**Fig. 1:**
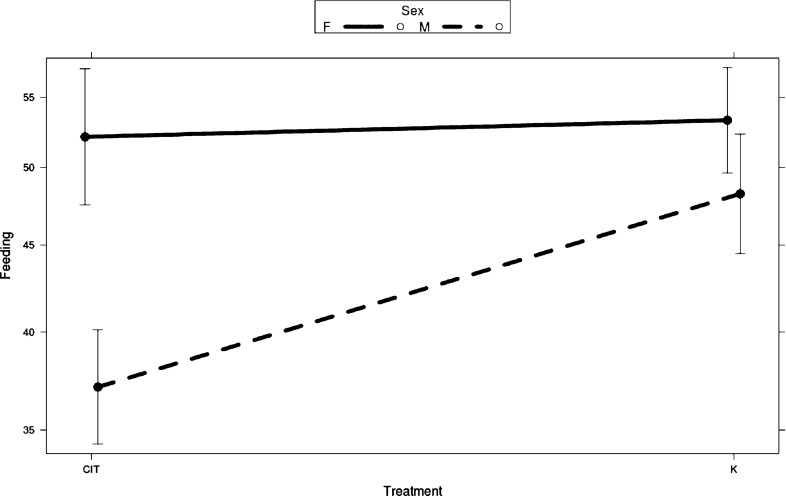
Results of the feeding test, showing the significant treatment*sex interaction. Bars represent 95% confidence intervals. The dataset included 23 females and 29 males

Sixty fish were originally included in the feeding trial, but eight fish had to be discarded from analysis because of camera malfunction and mortality. Of the remaining fish, 26 (17 males, 9 females) belonged to the exposed group and 26 (12 males, 14 females) to the control group. The initial analysis showed a sex*treatment interaction (*χ*^2^ = 6.13, *P* = 0.013; Fig. 1). In addition, all fixed factors in the model were statistically significant. When males and females were tested separately, there was a significant feeding suppression effect of treatment in the males (*χ*^2^ = 20.4, *P* < 0.001) but not in the females (*χ*^2^ = 0.91, *P* = 0.339). In addition, there was a significant effect of baseline for both males (*χ*^2^ = 90.9, *P *<0.001) and females (*χ*^2^ = 16.1, *P *<0.001). The individuals that had a high feeding rate before citalopram exposure continued to have a high feeding rate compared with other sticklebacks and vice versa. There was no Baseline*Treatment interaction in the original statistical model or in the separate analysis of either males (*χ*^2^ = 0.036, *P *=0.850) or females (*χ*^2^ = 0.12, *P *=0.730). The latency to feed was tested but yielded no significant effect of treatment (*χ*^2^ = 2.67, *P *=0.10), sex (*χ*^2^ = 0.65, *P *=0.42), or any interaction between fixed factors. Baseline was significant (*χ*^2^ = 6.06, *P *=0.014), meaning that baseline latency significantly influenced the results. Weight by the end of the experiment was not affected by either treatment (*χ*^2^ = 0.16, *P *=0.69) or sex (*χ*^2^ = 0.13, *P *=0.72) (final weights g, mean ± SD; Male controls: 1.09 ± 0.35, *n* = 12; Male citalopram: 1.15 ± 0.23, *n* = 17; Female controls: 1.07 ± 0.27, *n* = 14; Female citalopram: 1.24 ± 0.29, *n* = 9).

### Aggression

**Fig. 2:**
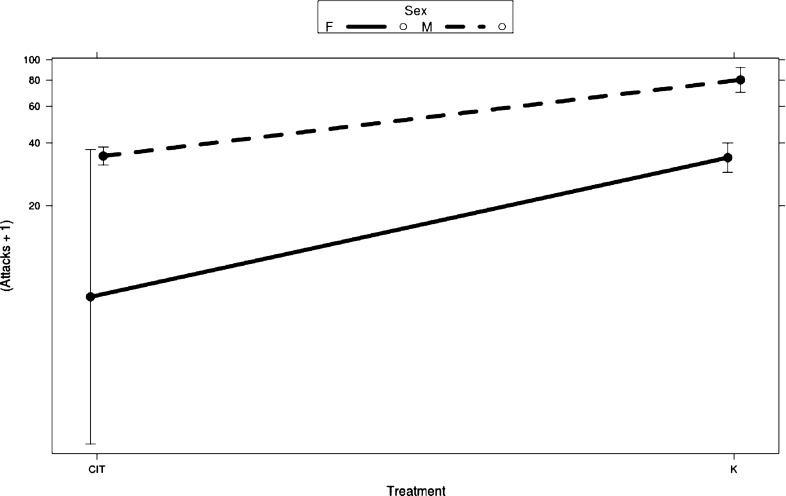
Results of the mirror test for aggression. Exposed fish are less aggressive than controls, and males are more aggressive than females. Bars represent 95% confidence intervals. The dataset included 9 females and 17 males

Sixty fish were tested for aggression in a mirror test, but only 15 (12 males, 3 females) exposed fish and 11 (5 males, 6 females) control fish could be included in the analysis. This was mostly because they did not come out of hiding, either during the baseline trial or after exposure, in time to discover the mirror and thus did not generate valid data. Fish that did discover the mirror generally showed a high level of aggression, and frequent attacks were noted. The mean number of attacks performed by the control fish was 59.9, whereas the exposed fish attacked the mirror 31.4 times. Aggressive behaviour was significantly affected by treatment (*χ*^2^ = 161.9, *P *<0.001; Fig. 2), sex (*χ*^2^ = 86.3, *P *<0.001; Fig. 2), and baseline value (*χ*^2^ = 58.8, *P *<0.001). Citalopram exposed fish were less aggressive than nonexposed fish, males were more aggressive than females, and those who were frequent attackers before exposure continued to be so. No interactions were noted between any of the fixed factors, although baseline*treatment was close to being statistically significant (*χ*^2^ = 3.29, *P *=0.07).

### Scototaxis

Sixty fish were originally included in the scototaxis study, but only 27 (17 males, 10 females) exposed fish and 27 (12 males, 15 females) control fish could be included due to mortality and camera malfunction. The mean of the total time spent in the white half of the aquarium was 249.2 s for the control fish and 213.9 s for the exposed fish. Almost without exception, the fish in the experiment first swam to the dark side of the aquarium and froze for some time before starting to explore first the dark side and eventually the white side of the aquarium. Statistical analysis yielded no significant effects of citalopram exposure or sex on time spent in the white half. However, a statistically significant sex*treatment interaction (*χ*^2^ = 5.1, *P *=0.024) was noted. Post-hoc testing showed that there was no significant effect in the males but that the exposed females spent significantly (*χ*^2^ = 5.02, *P *=0.050) less time in the white zone than the female controls. The mean latency to the first cross was 168.6 s for the control fish, whereas the mean latency for the exposed fish was 198.3 s. Again, there were no statistically significant effects of treatment or sex, but a statistically significant treatment*sex interaction (*χ*^2^ = 4.40, *P *=0.036). Post-hoc analysis showed no significant effect of treatment for either sex. The number of crossings to the white side was 10.6 for the control fish and 10.7 for the exposed fish. There was no significant effect of treatment or sex, and neither was there any significant treatment*sex interaction.

### Behavioural Syndromes

Correlations between aggression (*N* = 26, 15 exposed and 11 control) and feeding and between feeding (*N* = 52, 26 exposed and 26 control) and the “time spent on the white side” variable in the scototaxis test (*N* = 54, 27 exposed and 27 control) were examined both for the dataset as a whole and for the exposed and control subsets separately. In neither case was there any significant correlation. Correlation between aggression and total time in the white half in the scototaxis test also was examined both for the dataset as a whole, for exposed fish and controls separately and for males (*N* = 17) and females (*N* = 9) separately. In neither case was there any significant correlation.

### Water Samples

For economic reasons, not all samples were analysed. The samples analysed were taken at the beginning, at the middle, and at the end of the study. The measured concentration of citalopram in the water samples ranged from 350 to 380 ng/l (*n* = 4). In addition, citalopram was present in the control samples in low ng concentrations ranging from 1.5 to 1.7 ng/l (*n* = 2).

## Discussion

The current study showed, for the first time, a differential response to citalopram between male and female sticklebacks and that only male sticklebacks feeding behaviour was affected by citalopram exposure. A more pronounced effect from SSRI on male feeding behaviour compared with female was previously reported in zebrafish (*Danio rerio*) (Nielsen et al. 2019). Nielsen et al. (2019) used escitalopram, the active S-enantiomere of citalopram. To our knowledge, no other study has reported such differences between males and females. Although the reason for the sex difference observed in the current study and in Nielsen et al. (2019) is unknown, other effects of SSRIs, such as anxiolysis, have been shown to be sex-dependent in Endler guppy (*Poecilia wingei*) after exposure to citalopram (Olsén et al. 2014), the Eastern mosquitofish (*Gambusia holbrooki*) where fluoxetine provokes a sex-divergent response in freezing behaviour (Martin et al. 2017), and in rodents (Fernández-Guasti et al. 2017; Lebrón-Milad et al. 2013). In studies with rodents, there are mixed effects of SSRI exposure on sex difference in feeding and changes in weight (Currie et al. 2004; Hutchison et al. 2018). 5-HT is directly involved in regulation of feeding (Lee and Clifton 2010). It should be noted that the previously mentioned studies on rodents were not performed with citalopram as in the current study, but with fluoxetine, another SSRI. While citalopram and fluoxetine share the same mode of action, they differ in specificity for the 5-HT transporter (Owens et al. 2001). Several previous studies have found that SSRI exposure suppresses feeding in fish (Kellner et al. 2015; Mennigen et al. 2010; Weinberger and Klaper 2014) and other organisms (Falibene et al. 2012). This study confirms the findings of those studies for the population as a whole. The current study also shows a strong stringency over time in how many feeding strikes were performed by the individual sticklebacks, i.e., the individuals that performed many feeding-strikes in the baseline study continued to do so after exposure compared with the other fish in the study. This was true for both sexes and in both the exposed and control fish in the current study, indicating that the propensity to feed is a personality trait in the three-spine stickleback. Feeding as a personality trait has previously been demonstrated in other fish species (Silva et al. 2014; Vaz-Serrano et al. 2011).

The study of aggressive behaviour showed that citalopram attenuates aggression in the three-spine stickleback. Thus, the results of the current study confirms those of previous studies that have found SSRI-induced suppression of aggressive behaviour in various species, such as the Siamese fighting fish (Dzieweczynski and Hebert 2012; Kohlert et al. 2012), rainbow trout (Lepage et al. 2005), round goby (McCallum et al. 2017), bluehead wrasse (Perreault et al. 2003), and three-spine stickleback (Norton and Carreño Gutiérrez 2019). Holmberg and collaborators (2011) did not observe any change in aggression in rainbow trout fry after 6–7 days exposure to 1, 10, and 100 µg l^−1^ citalopram. Abbee-Lee and collaborators (2019) studied the stickleback behaviour in novel tank after exposure to 200 ng l ^−1^ of fluoxetine. The latency to swim was reduced after 18 days of exposure, but there were no effects on the other five behaviours recorded. Exposure to a dopamine receptor agonist, ropinirole (2500 ng l ^−1^), decreased the number of times the fish attacked a mirror. The exposures did not change the gene expressions of seven monoamine or stress-associated neurotransmitter genes. Because the actual concentrations of fluoxetine and ropinirole were not measured, the lack of effects could have been caused by low concentrations. In studies with citalopram, measured concentrations are much lower than the nominal concentrations (Kellner et al. 2015, 2016; present study). It also is important to measure concentrations of the studied pharmaceuticals in the tap water used (Porseryd et al. 2017; present study). Filby et al. (2010) did not observe any statistically significant changes in aggression in dominant zebrafish males after very short exposures: 1 h to 3 and 4.5 µg l ^−1^ of fluoxetine. However, blocking of the serotonin receptor HTR_1A_ with an antagonist (WAY100,635) increased aggression. The authors suggested that, in addition to the 5-HT pathway, other systems, such as the dopamine pathway, control aggression in zebrafish. Dahlbom et al. (2012) observed high aggressiveness in both zebrafish males and females in pairs with the same sex, and subordinate fish of both sexes had elevated hindbrain serotonergic activity (increased 5-HIAA/5-HT ratio; 5-HIAA is the metabolite of 5-HT). No differences in the dopaminergic system were observed between dominant and subordinate fish of both sexes. There were no differences between dominate and subordinates in the forebrain, but there were sex differences in dopamine levels and serotonergic activity. The same pattern with elevated serotonergic brain activity has been shown in subordinate individuals of Arctic charr (*Salvelinus alpinus*) (Winberg et al. 1991). In Arctic charr, social dominance can be induced by L-DOPA, the precursor of dopamine (Winberg and Nilsson 1992).

In the current study, males were more aggressive than females. Both males and females were at a sexually mature age but were not in spawning season and did not display nuptial colouring. Higher aggression in males than in females was previously noted in sexually mature three-spine stickleback (Bakker 1985) and other fish species (Davis, Harris, and Shelby 1974; Filby et al. 2010), but there are exceptions (Dahlbom et al. 2012; Ariyomo et al. 2013). In zebrafish, boldness and aggressiveness are heritable, but there are maternal influences on the offspring (Ariyomo et al. 2013). Maternal effects via the eggs have been shown in sticklebacks (Giesing et al. 2011) in studies of shoaling behaviour. The females had been exposed to and chased by a predator model, and stress hormones were transferred from the females into the eggs. In the present study, the individual sticklebacks showed strong stringency in behaviour regardless of citalopram treatment and sex, indicating that aggressiveness also is a personality trait in the three-spine stickleback.

The scototaxis study showed, somewhat surprisingly, that exposed females were less bold than control fish. This result should be interpreted with care, because it contradicts the expected anxiolytic effects of citalopram and was just barely statistically significant. The other variables in the scototaxis test yielded no significant interactions between fixed factors or any statistically significant effects, which was somewhat surprising. Sticklebacks that were exposed early during development to citalopram—from Day 2 to Day 30 after fertilization—and tested after 100 days in control water showed fewer crossings into the white zone (Kellner et al. 2018). They also were, against predictions, more aggressive and showed higher feeding frequency compared with control fish. These behavioural consequences are opposite to we have observed directly after citalopram exposures.

In the present study, we also examined the possibility of behavioural syndromes by testing for correlations between various behaviours. There was no significant correlation between feeding behaviour and aggression or time spent on the white side in the scototaxis test. The lack of correlation between feeding and time spent on the white side in the scototaxis test indicate that the propensity to feed is not related to boldness and lends no support to the existence of behavioural syndromes with the studied behaviours.

The citalopram concentrations in the samples were lower than nominal. This is a common observation when working with citalopram exposure in fish (Kellner et al. 2015, 2016) and may be caused by biodegradation (Evans et al. 2017), photodegradation (Evans et al. 2017), or adherence to particles in the water (Kwon and Armbrust 2008). Citalopram also was present in nanogram concentrations in the control samples. Because care was taken to prevent this kind of pollution in the lab, the authors believe that this citalopram pollution stems from the tap water used to house the fish. Similar pollution was found in another study using water from the same source (Porseryd et al. 2017). To the best knowledge of the authors, there is no record in literature of behavioural effects on fish of the citalopram levels present in the control samples, 1.5 and 1.7 ng/l. Low concentrations of pharmaceuticals have been detected in tap water for human consumption (Benotti et al. 2009).

Studies of differential responses to SSRI depending on personality in fish are few. Fior et al. (2018) found that shier and bolder zebrafish differed in their response to fluoxetine as measured by a novel object test. LePage et al. (2005) found that citalopram reduced aggression in rainbow trout but only for individuals with experience of being dominant. However, such differential responses have been reported in other organism groups, such as insects (Krams et al. 2018) and humans (Simmons and Allen 2011). While feeding behaviour and aggression could be pointed out as behaviours that persist over time in the three-spine stickleback and thus can be labelled personality traits, the current study failed to find any personality-dependent differences in the response to citalopram treatment. Furthermore, no evidence of behavioural syndromes was found. This may in part be due to the relatively few samples in the study of aggressive behaviour. Aggression and feeding behaviour were found to be sensitive to the relatively low citalopram concentrations measured in this study. Those are concentrations similar to or just above concentrations that have been found in aquatic environments (González Alonso et al. 2010). Aggression and feeding are ecologically important variables that affect survival and might affect food web composition (Hedgespeth et al. 2014). A higher level of perceived satiation suppresses predator inspection behaviours, risk taking (Godin et al. 1994), and therefore predation risk. On the other hand, it also suppresses growth and fitness. Weight was not affected in the current study. This may either be because the study time was quite short or because changes in weight were obscured by the fact that the fish were only weighed after the exposure period, so the effects on weight of the individual fish is unknown. Reduced weight as a result of SSRI exposure has previously been found in goldfish (Mennigen et al. 2009). It is likely that an SSRI-induced decrease in food intake has ecological implications, but such interpretations of the results are beyond the scope of this study. Perhaps the most interesting result in the present study is that males and females differed in their response to citalopram exposure regarding feeding behaviour and possibly in the scototaxis test. Whether this has any ecological significance beyond the general feeding suppressing effects needs further research. Because we know that citalopram has effect on feeding and aggression in the three-spine stickleback, one interesting next step would be to study competition for resources between exposed and unexposed fish.

## References

[CR1] Abbey-Lee RN, Kreshchenko A, Fernandez Sala X, Petkova I, Løvlie H (2019). Effects of monoamine manipulations on the personality and gene expression of three-spined sticklebacks. J Exp Biol.

[CR2] Ariyomo TO, Carter M, Watt PJ (2013). Heritability of boldness and aggressiveness in the zebrafish. Behav Genet.

[CR4] Bakker TCM (1985). Two-way selection for aggression in juvenile, female and male sticklebacks (*Gasterosteus aculeatus* L.), with some notes on hormonal factors. Behaviour.

[CR5] Bakker TCM (1994). Genetic correlations and the control of behaviour, exemplified by aggressiveness in sticklebacks. Adv Study Behav.

[CR6] Bates D, Maechler M, Bolker B, Walker S (2015). Linear mixed-effects models using Eigen. J Stat Softw.

[CR7] Bell AM (2005). Behavioural differences between individuals and two populations of stickleback (*Gasterosteus aculeatus*). J Evol Biol.

[CR8] Bell AM (2007). Future directions in behavioural syndromes research. Proc Biol Sci.

[CR9] Bell AM, Sih A (2007). Exposure to predation generates personality in three-spined stickleback (*Gasterosteus aculeatus*). Ecol Lett.

[CR10] Bell AM, Stamps JA (2004). Development of behavioural differences between individuals and populations of sticklebacks, *Gasterosteus aculeatus*. Anim Behav.

[CR11] Benotti MJ, Trenholm RA, Vanderford BJ, Holady JC, Stanford BD, Snyder SA (2009). Pharmaceuticals and endocrine disrupting compounds in U.S. drinking water. Environ Sci Technol.

[CR12] Brooks BW, Turner PK, Stanley JK, Weston JJ, Glidewell EA, Foran CM, Slattery M, La Point TW, Huggett DB (2003). Waterborne and sediment toxicity of fluoxetine to select organisms. Chemosphere.

[CR13] Caio M, de Brito TM, de Mattos Dias CAG, Gouveia A, Morato S (2010). Scototaxis as anxiety-like behavior in fish. Nat Protocols.

[CR14] Carere C, Eens M (2005). Unravelling animal personalities: how and why individuals consistently differ. Behaviour.

[CR15] Carter AJ, Feeney WE, Marshall HH, Cowlishaw G, Heinsohn R (2013). Animal personality: what are behavioural ecologists measuring?. Biol Rev.

[CR81] Chiffre A, Clérandeau C, Dwoinikoff C, Le Bihanic F, Budzinski H, Geret F, Cachot J (2016). Psychtropic drugs in mixture alter swiming behaviour of Japanese medaka (*Oryzias latipes*) larvae above environmental concentrations. Environ Sci Pollut Res.

[CR16] Clotfelter ED, O’Hare EP, McNitt MM, Carpenter RE, Summers CH (2007). Serotonin decreases aggression via 5-HT1A receptors in the fighting fish *Betta splendens*. Pharmacol Biochem Behav.

[CR17] Currie PJ, Braver M, Mirza A, Sricharoon K (2004). Sex differences in the reversal of fluoxetine-induced anorexia following raphe injections of 8-OH-DPAT. Psychopharmacology.

[CR18] Dahlbom SJ, Backström T, Lundstedt-Enkel K, Winberg S (2012). Aggression and monoamines: effects of sex and social rank in zebrafish (*Danio rerio*). Behav Brain Res.

[CR19] Davis RE, Harris C, Shelby J (1974). Sex differences in aggressivity and the effects of social isolation in the anabantoid fish, *Macropodus opercularis*. Behav Biol.

[CR20] De Pedro N, Pinillos ML, Valenciano AI, Alonso-Bedate M, Delgado MJ (1998). Inhibitory effect of serotonin on feeding behavior in goldfish: involvement of CRF. Peptides.

[CR21] Dingemanse NJ, Réale D (2005). Natural selection and animal personality. Behaviour.

[CR22] Dingemanse NJ, Wright J, Kazem AJN, Thomas DK, Hickling R, Dawnay N (2007). Behavioural syndromes differ between predictably between 12 populations of three-spined stickleback. J Anim Ecol.

[CR23] Dzieweczynski TL, Hebert OL (2012). Fluoxetine alters behavioral consistency of aggression and courtship in male Siamese fighting fish, *Betta splendens*. Physiol Behav.

[CR24] Dzieweczynski TL, Campbell BA, Kane JL (2016). Dose-dependent fluoxetine effects on boldness in male Siamese fighting fish. J Exp Biol.

[CR25] Egan RJ, Bergner CL, Hart PC, Cachat JM, Canavello PR, Elegante MF, Elkhayat SI (2009). Understanding behavioral and physiological phenotypes of stress and anxiety in zebrafish. Behav Brain Res.

[CR26] Eklöf JS, Sundblad G, Erlandsson M, Donadi S, Hansen JP, Klemens Eriksson B, Bergström U (2020). A spatial regime shift from predator to prey dominance in a large coastal ecosystem. Commun Biol.

[CR27] Evans S, Bagnall J, Kasprzyk-Hordern B (2017). Enantiomeric profiling of a chemically diverse mixture of chiral pharmaceuticals in urban water. Environ Pollut.

[CR28] Falibene A, Rössler W, Josens R (2012). Serotonin depresses feeding behaviour in ants. J Insect Physiol.

[CR29] Fernández-Guasti A, Olivares-Nazario M, Reyes R, Martínez-Mota L (2017). Sex and age differences in the antidepressant-like effect of fluoxetine in the forced swim test. Pharmacol Biochem Behav.

[CR30] Filby AL, Paull GC, Hickmore TFA, Tyler CR (2010). Unravelling the neurophysiological basis of aggression in a fish model. BMC Genom.

[CR31] Fior D, Dametto F, Fagundes M, Santos da Rosa JG, Sander de Abreu M, Koakoski G, Idalencio R, de Alcântara Barcellos HH, Piato A, Gil Barcellos LJ (2018). Divergent action of fluoxetine in zebrafish according to responsivity to novelty. Sci Rep.

[CR82] Gago-Ferrero P, Gros M, Ahrens L, Wiberg K (2017). Impact of on-site, small and large scale wastewater treatment facilities on levels and fate of pharmaceuticals, personal care products, artificial sweeternes, pesticides, and perfluoroalkyl substances in recipient waters. Science of the Total Environment.

[CR32] Giacomini AC, Murilo VV, Abreu S, Giacomini LV, Siebel AM, Zimerman FF, Rambo CL, Mocelin R, Bonan CD, Piato AL, Barcellos LJG (2016). Fluoxetine and diazepam acutely modulate stress induced-behavior. Behav Brain Res.

[CR33] Giesing ER, Suski CD, Warner RE, Bell AM (2011). Female sticklebacks transfer information via eggs: effects of maternal experience with predators on offspring. Proc R Soc B.

[CR34] Godin J-G, Shelley J, Crossman L (1994). Hunger-dependent predator inspection and foraging behaviours in the threespine stickleback (*Gasterosteus aculeatus*) under predation risk. Behavioral Ecol Sociobiol.

[CR35] González A, Catalá SM, Maroto RR, Rodríguez Gil JL, Gil de Miguel A, Valcárcel Y (2010). Pollution by psychoactive pharmaceuticals in the rivers of Madrid metropolitan area (Spain). Environ Int.

[CR36] Gosling SD (2001). From mice to men: what can we learn about personality from animal research?. Psychol Bull.

[CR37] Hedgespeth ML, Nilsson PA, Berglund O (2014). Ecological implications of altered fish foraging after exposure to an antidepressant pharmaceutical. Aquat Toxicol.

[CR38] Herborn KA, Macleod R, Miles WTS, Schofield ANB, Alexander L, Arnold KE (2010). Personality in captivity reflects personality in the wild. Anim Behav.

[CR39] Holmberg A, Fogel J, Albertsson E, Fick J, Brown JN, Paxéus N, Förlin L, Johnsson JI, Larsson DGJ (2011). Does waterborne citalopram affect the aggressive and sexual behaviour of rainbow trout and guppy?. J Hazard Mater.

[CR40] Huntingford FA (1976). The relationship between anti-predator behaviour and aggression among conspecifics in the three-spined stickleback, *Gasterosteus aculeatus*. Anim Behav.

[CR41] Hutchison SM, Mâsse LC, Pawluski JL, Oberlander TF (2018). Perinatal selective serotonin reuptake inhibitor (SSRI) effects on body weight at birth and beyond: a review of animal and human studies. Reprod Toxicol.

[CR42] Kellner M, Porseryd T, Porsch-Hällström I, Hansen SH, Olsén KH (2015). Environmentally relevant concentrations of citalopram partially inhibit feeding in the three-spine stickleback (*Gasterosteus aculeatus*). Aquat Toxicol.

[CR43] Kellner M, Porseryd T, Hallgren S, Porsch-Hällström I, Hansen SH, Olsén KH (2016). Waterborne citalopram has anxiolytic effects and increases locomotor activity in the three-spine stickleback (*Gasterosteus aculeatus*). Aquat Toxicol.

[CR44] Kellner M, Porseryd T, Porsch-Hällström I, Borg B, Roufidou C, Olsén KH (2018). Developmental exposure to the SSRI citalopram causes long-lasting behavioural effects in the three-spined stickleback (*Gasterosteus aculeatus*). Ecotoxicology.

[CR45] Kohlert JG, Mangan BP, Kodra C, Drako L, Long E, Simpson H (2012). Decreased aggressive and locomotor behaviors in *Betta splendens* after exposure to fluoxetine. Psychol Rep.

[CR46] Krams I, Trakimas G, Kecko S, Elferts D, Krams R, Luoto S, Rantala MJ (2018). Linking organismal growth, coping styles, stress reactivity, and metabolism via responses against a selective serotonin reuptake inhibitor in an insect. Sci Rep.

[CR47] Kwon JW, Armbrust KL (2008). Aqueous solubility, n-octanol–water partition coefficient, and sorption of five selective serotonin reuptake inhibitors to sediments and soils. Bull Environ Contam Toxicol.

[CR48] Lebrón-Milad K, Tsareva A, Ahmed N, Milad MR (2013). Sex differences and estrous cycle in female rats interact with the effects of fluoxetine treatment on fear extinction. Behav Brain Res.

[CR49] Lee MD, Clifton PG (2010). Role of the serotonergic system in appetite and ingestion control. Handbook of the behavioural neurobiology of serotonin.

[CR50] Lepage O, Larson ET, Mayer I, Winberg S (2005). Serotonin, but not melatonin, plays a role in shaping dominant–subordinate relationships and aggression in rainbow trout. Hormones Behav.

[CR51] Lillesaar C (2011). The serotonergic system in fish. J Chem Neuroanat.

[CR52] Martin JM, Saaristo M, Bertram MG, Lewis PJ, Coggan TL, Clarke BO, Wong BBM (2017). The psychoactive pollutant fluoxetine compromises antipredator behaviour in fish. Environ Pollut.

[CR53] Martins EP, Bhat A (2014). Population-level personalities in zebrafish: aggression-boldness across bot not within populations. Behav Ecol.

[CR54] McCallum ES, Bose APH, Warriner TR, Balshine S (2017). An evaluation of behavioural endpoints: the pharmaceutical pollutant fluoxetine decreases aggression across multiple contexts in round goby (*Neogobius melanostomus)*. Chemosphere.

[CR55] Mennigen JA, Harris EA, Chang JP, Moon TW, Trudeau VL (2009). Fluoxetine affects weight gain and expression of feeding peptides in the female goldfish brain. Regul Peptides.

[CR56] Mennigen JA, Sassine J, Trudeau VL, Moon TW (2010). Waterborne fluoxetine disrupts feeding and energy metabolism in the goldfish *Carassius auratus*. Aquat Toxicol.

[CR57] Nielsen SV, Frausing M, Guldhammer Henriksen P, Beedholm K, Baatrup E (2019). The psychoactive drug escitalopram affects foraging behavior in zebrafish (*Danio rerio*). Environ Toxicol Chem.

[CR58] Niemelä PT, Dingemanse NJ (2014). Artificial environments and the study of adaptive personalities. Trends Ecol Evol.

[CR59] Norton WHJ, Carreño Gutiérrez H (2019). The three-spined stickleback as a model for behavioural neuroscience. PLoS ONE.

[CR60] Nowicki M, Tran S, Muraleetharan A, Markovic S, Gerlai R (2014). Serotonin antagonists induce anxiolytic and anxiogenic.like behavior in zebrafish in a receptor-subtype dependent manner. Pharmacol Biochem Behav.

[CR61] Olsén KH, Ask K, Olsén H, Porsch-Hällström I, Hallgren S (2014). Effects of the SSRI citalopram on behaviours connected to stress and reproduction in Endler guppy, *Poecilia wingei*. Aquat Toxicol.

[CR62] Ortega VA, Lovejoy DA, Bernier NJ (2013). Appetite-suppressing effects and interactions of centrally administered corticotropin-releasing factor, urotensin I and serotonin in rainbow trout (*Oncorhynchus mykiss*). Front Neurosci.

[CR63] Øverli Ø, Páll M, Borg B, Jobling M, Winberg S (2001). Effects of *Schistocephalus solidus* infection on brain monoaminergic activity in female three-spined sticklebacks *Gasterosteus aculeatus*. Proc R Soc B Biol Sci.

[CR64] Owens MJ, Knight DL, Nemeroff CB (2001). Second-generation SSRIs: human monoamine transporter binding profile of escitalopram and R-fluoxetine. Biol Psychiat.

[CR65] Perreault H, Semsar K, Godwin J (2003). Fluoxetine treatment decreases territorial aggression in a coral reef fish. Physiol Behav.

[CR66] Porseryd T, Kellner M, Neyhanian Caspillo N, Volkova K, Elabbas L, Ullah S, Olsén H, Dinnétz P, Porsch Hällström I (2017). Combinatory effects of low concentrations of 17α-etinylestradiol and citalopram on nonreproductive behavior in adult zebrafish (*Danio rerio*). Aquat Toxicol.

[CR83] Rozenblit-Susan S, Chapnik N, Genzer Y, Froy O (2016). Serotonin suppresses food anticipatory activity and synchronize the food-entrainable oscillator during time-restricted feeding. Behavioural Brain Research.

[CR67] Schjolden J, Backstrom T, Pulman K, Pottinger T, Winberg S (2005). Divergence in behavioural responses to stress in two strains of rainbow trout (*Oncorhynchus mykiss*) with contrasting stress responsiveness. Hormones Behav.

[CR69] Sih A, Mathot KJ, Moirón M, Montiglio P-O, Wolf M, Dingemanse NJ (2015). Animal personality and state-behaviour feedbacks: a review and guide for empiricists. Trend Ecol Evol.

[CR70] Silva PIM, Martins CIM, Höglund E, Gjøen HM, Øverli Ø (2014). Feeding motivation as a personality trait in Nile tilapia (*Oreochromis niloticus*): role of serotonergic neurotransmission. Fish Physiol Biochem.

[CR71] Simmons JG, Allen NB (2011). Mood and personality effects in healthy participants after chronic administration of sertraline. J Affect Disorders.

[CR100] Stamps JA, Biro PA (2016). Personality and individual differences in plasticity. Curr Opin Behav Sci.

[CR73] R core team (2015) R: a language and environment for statistical computing. 2015. http://www.R-project.org/

[CR74] Thörnqvist P-O, Höglund E, Winberg S (2015). Natural selection constrains personality and brain gene expression differences in Atlantic salmon (*Salmo salar*). J Exp Biol.

[CR75] Vaz-Serrano J, Ruiz-Gomez ML, Gjøen HM, Skov PV, Huntingford FA, Øverli Ø, Höglund E (2011). Consistent boldness behaviour in early emerging fry of domesticated Atlantic salmon (*Salmo salar*): decoupling of behavioural and physiological traits of the proactive stress coping style. Physiol Behav.

[CR76] Voigt J-P, Fink H (2015). Serotonin controlling feeding and satiety. Behav Brain Res.

[CR77] Weinberger J, Klaper R (2014). Environmental concentrations of the selective serotonin reuptake inhibitor fluoxetine impact specific behaviors involved in reproduction, feeding and predator avoidance in the fish *Pimephales promelas* (fathead minnow). Aquat Toxicol.

[CR78] Weinstein T, Capitanio J, Gosling S, John OP, Robins RW, Pervi LA (2008). Personality in animals. Handbook of personality: theory and research.

[CR79] Winberg S, Nilsson GE (1992). Induction of social-dominance by L-DOPA treatment in Arctic charr. NeuroReport.

[CR80] Winberg S, Nilsson GE, Olsén KH (1991). Social rank and brain levels in monoamines and monoamine metabolites in Arctic harr (*Salvelinus alpinus* L.) are socially induced. J Comp Physiol A Sens Neural Behav Evolut.

